# Exploring whether primary care networks can contribute to the national goal of reducing health inequalities: a mixed-methods study

**DOI:** 10.3399/BJGP.2023.0258

**Published:** 2024-04-16

**Authors:** Lynsey Warwick-Giles, Joseph Hutchinson, Kath Checkland, Jonathan Hammond, Donna Bramwell, Simon Bailey, Matt Sutton

**Affiliations:** Centre for Primary Care and Health Services Research, University of Manchester, Manchester.; Centre for Primary Care and Health Services Research, University of Manchester, Manchester.; Centre for Primary Care and Health Services Research, University of Manchester, Manchester.; Centre for Primary Care and Health Services Research, University of Manchester, Manchester.; Centre for Primary Care and Health Services Research, University of Manchester, Manchester.; Centre for Health Services Studies, University of Kent, Canterbury.; Centre for Primary Care and Health Services Research, University of Manchester, Manchester.

**Keywords:** health inequalities, mixed methods, policy, primary care networks, primary health care

## Abstract

**Background:**

Significant health inequalities exist in England. Primary care networks (PCNs), comprised of GP practices, were introduced in England in 2019 with funding linked to membership. PCNs have been tasked with tackling health inequalities.

**Aim:**

To consider how the design and introduction of PCNs might influence their ability to tackle health inequalities.

**Design and setting:**

A sequential mixed-methods study of PCNs in England.

**Method:**

Linear regression of annual PCN-allocated funding per workload-weighted patient on income deprivation score from 2019–2023 was used. Qualitative interviews and observations of PCNs and PCN staff were undertaken across seven PCN sites in England (July 2020–March 2022).

**Results:**

Across 1243 networks in 2019–2020, a 10% higher level of income deprivation resulted in £0.31 (95% confidence interval [CI] = £0.25 to £0.37), 4.50%, less funding per weighted patient. In 2022–2023, the same difference in deprivation resulted in £0.16 (95% CI = £0.11 to £0.21), 0.60%, more funding. Qualitative interviews highlighted that, although there were requirements for PCNs to tackle health inequalities, the policy design, and PCN internal relationships and maturity, shaped and sometimes restricted how PCNs approached this task locally.

**Conclusion:**

Allocated PCN funding has become more pro-poor over time, suggesting that the need to account for deprivation within funding models is understood by policymakers. The following additional approaches have been highlighted that could support PCNs to tackle inequalities: better management support; encouragement and support to redistribute funding internally to support practices serving more deprived populations; and greater specificity in service requirements.

## Introduction

Health inequalities is a commonly used, but often poorly defined, concept referring to differences in experiences and outcomes of health and illness across populations. These differences are driven by multiple factors, including socioeconomic and social influences, as well as by inequalities in service provision.^1^ Important inequalities include differences in morbidity, mortality, health status, access to care, and quality of care received, and have been clearly documented over many years, most recently in relation to outcomes associated with the COVID-19 pandemic.^2^^–^^7^

Recent health policy in England has emphasised the role of healthcare services in reducing inequalities. The *NHS Long Term Plan* has set out what is described as a *‘concerted and systematic approach to reducing health inequalities’*, with associated actions.^8^ Primary care has an important role to play, with studies over many years demonstrating an inverse care law by which care is least available to those populations that need it most.^9^ It is therefore important to consider how new policies in this context impact on inequality. This paper has explored the implementation of primary care networks (PCNs) in England and has considered their potential impact on health inequalities.

### Primary care networks

English general practice is usually provided by partnerships of primary care physicians (GPs), according to a General Medical Services contract. The contract is held between the NHS and independent GP practices, outlining both mandatory and additional services that GP practices must provide to their patients. Additional work can be commissioned via an add-on voluntary contract known as directed enhanced services (DES). In 2019, this mechanism was used to encourage groups of practices to work together as PCNs. Covering a patient population of approximately 30 000–50 000, the DES provides PCNs with extra resources in exchange for the delivery of additional services.^10^ These included seven new ‘service specifications’, alongside extended hours appointments.

**Table:** How this fits in

Primary care networks (PCNs) are an important policy development in English primary care, with an additional contract supporting practices to work collaboratively. Policymakers intend that they will tackle local health inequalities. This research has suggested that there is potential for PCNs to achieve this aim, but the networks will require the following: continued weighting of funding formulae to account for deprivation; redistribution of funds and other resources internally to support the most deprived practices; managerial support, particularly for PCNs with deprived populations; and realistic and achievable targets for PCN action.

PCNs represent the latest in a long history of policies designed to encourage and incentivise individual GP practices in England to work more closely together. Such policies are underpinned by an assumption that individual GP practices are too small to deliver modern primary care services alone, and previous policy examples include the following: fundholding and total purchasing pilots; primary care groups and trusts; practice-based commissioning; and clinical commissioning groups (CCGs). Each of these initiatives involved funding and incentives to support practices to work collectively, although the exact goals and approaches varied between schemes. Some focused largely on service provision by practices, while others also involved the engagement of GPs in wider issues of local service planning and commissioning. As initially established, the focus of PCNs is on collective provision of services, but those responsible for the policy also see a wider role for them in representing primary care in local and regional decision making about service provision.

The funding provided to PCNs is multifaceted (Table 1). Some payments are intended to support infrastructure, including a so-called ‘participation payment’ and funding to pay a clinical director. The most significant funding (approximately 50% of the total) is associated with the Additional Roles Reimbursement Scheme (ARRS), which reimburses networks for the salaries of a broad range of additional staff, including social prescribing link workers, mental health workers, physiotherapists, pharmacists, and physician associates. Finally, PCNs can earn incentive payments, via the Investment and Impact Fund. Since the present study, the PCN contract has been altered to include the capacity and access fund, which provides additional funding to PCNs to improve access for their patients.^11^

**Table 1.** Funding streams in the PCN directed enhanced service per financial year. Details are as per the contract with changes made mid-year not included (for example, COVID-19 vaccination programme)

**Funding stream**	**2019–2020 (pro-rata as** **payments started in July 2019)**	**2020–2021**	**2021–2022**	**2022–2023**
Network participation payment	£1.761 per contractor-weighted patient (Carr-Hill adjusted)	£1.761 per contractor-weighted patient	£1.761 per contractor-weighted patient	£1.761 per contractor-weighted patient
Clinical director	£0.514 per registered patient (unweighted)	£0.722 per registered patient	£0.736 per registered patient	£0.736 per registered patient
Core PCN funding	£1.50 per registered patient	£1.50 per registered patient	£1.50 per registered patient	£1.50 per registered patient
Additional Roles Reimbursement Scheme (variable)	<100 000 1 × WTE clinical pharmacist (maximum £37 810) 1 × WTE social prescribing link worker (maximum £34 113) Practices >100 000 can claim 1 further WTE per staff category for every 50 000 patients	£7.131 per PCN contractor-weighted population	£12.314 per PCN contractor-weighted population	£16.696 per PCN contractor-weighted population
Extended hours	£1.45 per registered patient	£1.45 per registered patient	£1.44 per registered patient	£0.720 per registered patient
Enhanced access				£3.764 per PCN-adjusted population (CCG allocation formula)
PCN support payment		£0.27 per PCN contractor-weighted population	£1.029 per PCN contractor-weighted population	
PCN capacity and access support payment				£0.602 per PCN-adjusted population
PCN leadership and management			£0.707 per PCN-adjusted population	£0.699 per PCN-adjusted population
*Care home premium (variable)*		£60 per bed	*£120 per bed*	*£120 per bed*
*Investment and Impact Fund (variable)*		*194 points. £111 per point*	*389 points. £200 per point (qualitative and quantitative; binary, standard, or improvement)*	*989 points. £200 per point (qualitative and quantitative; binary, standard, improvement, or composite)*

*Contractor-weighted list size = sum of Carr-Hill adjusted patient list from constituent practices. PCN-adjusted population = CCG primary medical care allocation formula. CCG = clinical commissioning group. PCN = primary care network. WTE = whole-time equivalent.*

It is an explicit aim of the PCN DES that the policy should contribute to the reduction in observed health inequalities. There are three potential mechanisms for achieving this aim. First, the policy offers funding to groups of practices, which is at least partially weighted to account for deprivation. Second, the policy directly requires specific activity relevant to inequalities. This is represented by a service specification requiring PCNs to develop a plan to tackle a locally important inequality. Third, there is a more indirect expectation that the collective activity led by a PCN health inequalities lead may catalyse more general changes in service delivery that could act to reduce inequalities.^12^ While policy documents do not explicitly consider how this might be achieved, it is expected that working together will encourage a more supportive environment within general practice. It is possible that this might, for example, lead to the internal redistribution of resources or support to help practices serving more deprived populations.

In this paper, the factors affecting the operation of these mechanisms are considered, to better understand how the policy could be optimised to meet its aims regarding health inequalities. Early analysis of the contract identified potential concerns about the distribution of resources, suggesting that known health inequalities were not fully reflected in the formulae used to determine funding.^13^ Here this analysis is extended, using a mixed-methods approach to explore the policy and its implementation.

## Method

This paper presents findings from a longitudinal mixed-methods project running between July 2019 and July 2022. This comprised policymaker and stakeholder interviews,^14^ telephone interviews with CCGs,^15^ qualitative case studies of PCNs, and quantitative analysis of PCN allocated funding. CCGs were established in 2012 as the statutory NHS bodies responsible for planning local services for their local population. At the time of the initial study, they retained responsibility for supporting the establishment of PCNs, although they have since been abolished. The initial qualitative data collection raised some queries as to whether the funding provided to PCNs was sufficiently adjusted to take account of deprivation and inequalities. This led to a quantitative analysis of the various funding mechanisms, the results of which fed into further qualitative data collection, exploring the factors affecting PCNs’ ability to use the funding provided to tackle inequalities.

### Quantitative contract analysis

To consider the contract design, the stated funding formulae used in the 2019–2020, 2020–2021, 2021–2022, and 2022–2023 DESs were focused on. The funding this provided was estimated and how this varied by deprivation after accounting for need was analysed. This represents the PCN contract before winter 2022, when the capacity and access fund was introduced.

### Data sources

#### Primary care network sample and population data

A full list of the 1255 PCNs and 6531 GP practices open on 1 January 2022 was gathered from NHS England (NHSE), with their unweighted and adjusted populations.^16^ NHSE is the organisation responsible for healthcare planning and delivery in England, with oversight from the government’s Department of Health and Social Care. All but 70 GP practices were already aligned to a network. These practices were identified in NHSE organisational data service, with seven practices subsequently aligned to a network, with the remaining (*n* = 63) not signed up to the network DES. The unweighted and adjusted populations of the subsequently aligned practices were added to their corresponding network to get the final unweighted and adjusted populations. The unweighted population refers to the PCN’s raw population. PCNs will differ in how much care their population needs and the associated costs, such as owing to different amounts of morbidity. These differences are accounted for through weighting (adjusting) the raw population.

The PCN DES uses two adjustments. The CCG allocation formula is used to model differences in need and associated workload between different CCGs.^17^ This is based on the registered population’s age–sex profile, health inequalities, new registrations, and rurality.^17^ This formula is applied to create the PCN-adjusted population. The contractor-weighted list size differs from this as it is the sum of the constituent practices’ weighted list sizes, which uses the Carr-Hill formula. This adjusts for the practice’s age–sex profile, additional needs, list turnover, market forces, and rurality.

The adjusted population is only available in January 2022, while the contractor population is available in March 2022. To account for differences in the population sizes between these time points, the March 2022 contractor weighting for each network was calculated in March 2022 by dividing the contractor-weighted population by the unweighted population (ranging from 0.63–1.39) and applied to the January 2022 unweighted network population, creating the contractor-weighted population in January 2022.

#### Stated funding formulae

Unfortunately, owing to missing data, it was impossible to use the payments that networks actually received. The allocated funding for each PCN for each contract year was calculated. The stated funding streams and formula for each year (2019–2020, 2020–2021, 2021–2022, and 2022–2023) of the PCN DES are detailed in Table 1.^10^^,^^18^^–^^20^ For the ARRS, the maximum amount of funding a network could be reimbursed was calculated. However, the amount actually reimbursed depended on network recruitment. The Impact and Investment Fund was excluded from the calculation as it is still unclear the extent to which it will catalyse activity in networks, which is integral to a pay-for-performance scheme and difficult to estimate.^21^^–^^23^ Similarly, the care home premium was excluded as data on number of care home beds per network are not available. Contract changes mid-year, such as funding for the capacity and access fund, were not included.

#### Average network income deprivation score

Income deprivation score is a continuous measure of the proportion of the population who receive benefits from the state on the grounds of low income. The larger the value the more deprived the population. This was gathered at practice level by combining the 2019 Office for National Statistics indices of deprivation data and 2020 lower-level super output area data.^24^ The PCN’s income deprivation score was then calculated as the sum of its constituent practices’ deprivation scores, relative to the population size of the practice.

#### Need adjustment

As the deprivation of a population increases, the authors expected the need and associated costs for care to increase relative to the population size. As such, to avoid reinforcing inequality, it was important that the funding formula at least accounted for this difference. To control for this, a needs adjustment was performed using the Carr-Hill formula. There are multiple approaches to control for this difference, such as the Carr-Hill formula (capitation adjustment), as well as age and sex-specific consultation rates, and population mortality and morbidity, with no universally agreed approach. Pre-existing English general practice funding is adjusted for need (workload adjustment) by the Carr-Hill formula. As such, need differences were adjusted for using this formula. This means it could be analysed whether the stated PCN funding formula has greater adjustment for deprivation relative to the existing adjustment used for general practice funding.

### Statistical analysis

Statistical analysis was conducted in R Studio (version 2022.07.2). Twelve networks (0.96%) had incomplete contractor-weighted list sizes, which were excluded from the analysis. Summary statistics were calculated for all variables. Linear regression was used to analyse the relationship between the PCN-unweighted, contractor-weighted, and adjusted populations, funding per contract year and funding per weighted patient by the network’s income deprivation score.

### Qualitative primary care network case studies

The factors affecting the ability of PCNs to tackle inequalities were explored in qualitative case studies of seven PCNs. The case studies were selected to capture heterogeneity of PCNs including size, population demographics, and geographical location (Table 2).

**Table 2.** Description of PCN case-study sites

	**PCN A**	**PCN B2**	**PCN B3**	**PCN C**	**PCN D**	**PCN E1**	**PCN E2**
GP member practices	10–15	15–20	10–15	5–10	5–10	5–10	5–10
Patient population	60 000–70 000	90 000–100 000	70 000–80 000	50 000–60 000	80 000–90 000	30 000–40 000	30 000–40 000
Population deprivation	High	Mixed	High	Mixed	Mixed	High	High
Collaboration history	Mixed collaborative history (some new practices added to the existing GP practice group to form the PCN)	Mixed collaborative history (some practices had worked closely together)	Limited collaboration	Loose collaboration links through previous CCG initiatives	Strong collaborative history	Practical collaborative history through previous national initiatives	Practical collaborative history through previous national initiatives

*CCG = clinical commissioning group. PCN = primary care network.*

Ninety-one qualitative interviews and approximately 87 hours of meeting (for example, PCN leadership team or PCN member meetings) observations were conducted by LWG (female), DB (female), JHa (male), and SB (male). All researchers have extensive qualitative research experience and did not know the participants before recruitment.

Sites were recruited based on the telephone interviews with CCGs and contact was made for interviews and observations via email. All sites were recruited using a project information sheet that outlined all the information about the research project, what taking part would entail, and the ethical considerations — for example, consent and anonymity. Interview participants were selected using purposive and snowball sampling. Information was provided by the main PCN contact or people were identified during meeting observations. No participants who were contacted declined to take part.

All interviews and observations took place via Microsoft Teams or Zoom because of COVID-19 pandemic working restrictions. Most participants were either working from their home or were within their GP practice. No other people were present other than the researcher and the participant. The sample comprised GPs, commissioners, ARRS staff, and NHS managers.

All interviews followed a topic guide, which was developed based on engagement with relevant policy documentation, academic literature, and the authors’ knowledge of primary care organisation and policy. All interviews were audio-recorded, and field notes were taken when observing meetings. Interviews lasted 1 hour on average and observations lasted 2 hours on average. The research team stopped collecting data when data saturation was reached — that is, no new themes were being discussed by participants. All field notes were typed up by the researchers and the interviews were transcribed by an independent company. None of the transcriptions were checked by participants.

A framework analysis method was employed.^25^ All data were coded and analysed by LWG, JHa, DB, and SB using NVivo (version 12). The coding framework was developed iteratively by the research team members. A deductive and inductive approach was taken, whereby some themes were developed before data collection (based on existing literature) and others were derived from the data. Initial findings were presented to a national PCN membership organisation to ensure that they had strong face validity with those involved in implementing the PCN policy.

Qualitative and quantitative researchers worked collaboratively, with insights from the quantitative work feeding into subsequent rounds of qualitative interviewing.

## Results

### The relationship between funding received and population deprivation

A final sample of 1243 networks was included with a mean PCN-registered list size of 48 927 (10% and 90% percentiles: 31 194, 69 988). Summary statistics are detailed in Table 3 and Table 4. Mean network income deprivation score was 0.129 (10% and 90% percentiles: 0.063, 0.213). Mean PCN contractor-weighted list size was 48 905 (10% and 90% percentiles: 30 921, 71 333) with mean PCN adjusted population 48 903 (10% and 90% percentiles: 30 427, 71 589). Mean estimated funding to PCN increased each contact year from £327 531 (10% and 90% percentiles: £235 308, £438 213) in 2019–2020 to £1 294 476 (10% and 90% percentiles: £818 675, £1 878 790) in 2022–2023. Similarly funding per weighted patient increased from £6.90 (10% and 90% percentiles: £6.10, £7.73) to £26.49 (£10% and 90% percentiles: 25.85, £27.07) in the same time period.

**Table 3.** Summary statistics on the population and deprivation for 1243 PCNs 2019–2023

**Variable**	**Mean**	**95% confidence interval of the mean**	**10% and 90% percentile**	**Standard deviation**
PCN-registered list size	48 927	47 813.27 to 50 042.32	31 194.4, 69 987.6	20 028.75
PCN contractor-weighted list size (Carr-Hill)	48 905	47 769.77 to 50 040.46	30 921.22, 71 333.37	20 402.89
PCN-adjusted population (CCG allocation formula)	48 903	47 763.27 to 50 042.59	30 427.19, 71 588.79	20 480.46
Income deprivation score	0.129	0.125 to 0.132	0.063, 0.213	0.06

*CCG = clinical commissioning group. PCN = primary care network.*

**Table 4.** Mean, confidence interval of the mean, 10% and 90% percentiles, and standard deviation of the estimated allocated funding per PCN and weighted patient 2019–2023

	**2019–2020**		**2020–2021**		**2021–2022**		**2022–2023**	
	**Estimated funding per PCN**	**Funding per weighted patient**	**Estimated funding per PCN**	**Funding per weighted patient**	**Estimated funding per PCN**	**Funding per weighted patient**	**Estimated funding per PCN**	**Funding per weighted patient**
Mean (£)	327 531	6.90	627 732	12.86	952 851.3	19.50	1 294 476	26.49
95% confidence interval (£)	321 702 to 333 360	6.86 to 6.93	613 307 to 642 157	12.84 to 12.87	930 893 to 974 810	19.48 to 19.52	1 264 586 to 1 324 367	26.46 to 26.51
10% and 90% percentile (£)	235 308, 438 213	6.10, 7.73	400 140, 906 540	12.45, 13.25	605 387, 1 384 073	19.06, 19.91	818 675, 1 878 790	25.85, 27.07
Standard deviation	104 748.8	0.65	259 227.8	0.33	394 611.1	0.35	537 151.7	0.50

*PCN = primary care network.*

Results of the linear regression are in Supplementary Table S1. A 10% (0.1) higher average network income deprivation score resulted in 3283 (95% confidence interval [CI] = 1368 to 5198), 5260 (95% CI = 3322 to 7197), and 7952 (95% CI = 6035 to 9868) more patients for unweighted, contractor-weighted, and adjusted populations, respectively. This indicates that networks in more deprived areas are larger, with greater weighting for income deprivation with the adjusted compared with contractor-weighted populations.

**Figure 1.**
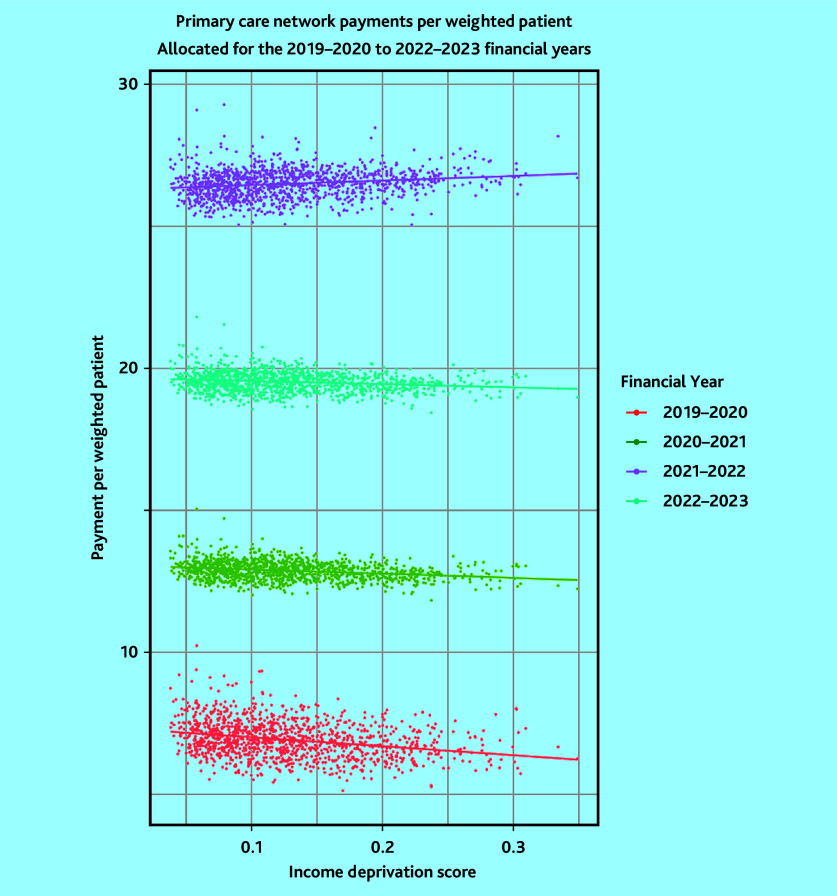
Scatterplot of payments per weighted patient by income deprivation per primary care network contract year 2019–2020 to 2022–2023.

When analysing how stated funding changes with deprivation relative to healthcare need, a gradual change over the four-contract years was identified. A 10% increase in deprivation resulted in £0.31 (95% CI = £0.37 to £0.25 less funding per weighted patient in 2019–2020, but in 2022–2023 the same increase in deprivation resulted in £0.16 (95% CI = £0.11 to £0.21) more funding per weighted patient (Table 5). This has indicated that the stated formula for PCNs provides greater weighting for deprivation than the existing adjustment used in the general practice global sum. Scatterplots of this relationship are shown in Figure 1.

**Table 5.** Univariate regression of funding per weighted patient on income deprivation score from 2019–2023. Results show the change from a 10% (0.1) increase in income deprivation score (more deprived)

	**2019–2020**	**2020–2021**	**2021–2022**	**2022–2023**
	**Funding per weighted patient (£)**	**Funding per weighted patient (£)**	**Funding per weighted patient (£)**	**Funding per weighted patient (£)**
β coefficient	−0.31	−0.15	−0.11	0.16
95% confidence interval	−0.37 to −0.25	−0.18 to −0.12	−0.14 to −0.08	0.11 to 0.21
*P-*value	<0.05	<0.05	<0.05	<0.05

### Direct requirements to tackle inequalities

The contract includes a service specification that requires PCNs to formulate a co-produced plan to tackle an important locally identified inequality. While this was generally welcomed by case study PCNs, it was also seen as potentially daunting. For example, in Site C, the membership expressed concerns that *‘the ask’* of PCNs was too great.

Health inequalities lead*: ‘PCNs have been asked to engage with people who are experiencing health inequalities, to co-design with them and implement an intervention … We know that in* [Area X] *that there are issues with air pollution, obesity, higher alcohol and drug misuse, and loneliness in the elderly. We have possible actions of looking at healthy families, engagement with the homeless, promote existing services.*[…]*. The priority we focus on will be the one that has the most votes.’*

Practice manager 1*: ‘I agree with the points. These are all obstacles that the PCN is facing. I do wonder how we are supposed to tackle some of them …’* (Site C, PCN meeting, 250122)

As illustrated here, the PCNs in the study were aware of the inequalities that their patient populations face. However, they also told us that, given the wider societal factors underpinning those issues, including housing, education, employment, and so on, they were concerned that, as healthcare providers, they had limited levers with which to tackle the problems that they saw. They were keen to engage with this agenda but would have welcomed more specific guidance and support.

### Indirect impact: collaboration as mechanism to reduce local inequalities

The study noted considerable variation in many aspects of PCN operation. These included variation in size, populations they serve, the development of internal relationships, and the types of practices within PCNs.^26^ Therefore, the extent to which the policy will enable collective working, which acts to reduce inequalities, is also likely to be variable. Several factors of relevance to this potential mechanism were identified, some related to the characteristics of different PCNs and some arising out of the design of the policy itself.

### Primary care network characteristics

Each PCN had the freedom to develop local arrangements for working together. This proved to be easier for some groups than others. In Site A, there were a number of different operating models of general practice within the PCN membership, with practices working within a number of different contract models collaborating together. The diverse operating models influenced how individual practices viewed their purpose, how they engaged with their patient population, and how they wished to provide services. One practice struggled to engage with the wider PCN owing to the circumstances by which the practice had joined the PCN. The relationship did not develop organically; instead, the practice joined the PCN under the instruction of the local CCG:

*‘We feel as though we’re a bit of an oddity around the place and they* [the clinical commissioning group] *weren’t sure where to put us, so they put us with that team. And then that team turned out to have existing relationships of neighbours and they’ve got on and run it as best they can since then. That’s how it feels.’*

(N590yb-GP, Site A)

In addition, the existing practices within the PCN had worked together in the past, which had allowed relationships and ways of working to develop over time. This made it more difficult for the practice joining later; for example, organisational structures, ways of working, and leadership had previously been established, which made it more challenging for new practices to find their place within the PCN. In addition to the varied general practice models, there was also considerable variation in practice list sizes across the PCN membership. Differing practice sizes meant that there were different staff numbers across practices, affecting how practices engaged with the PCN agenda. Smaller practices have fewer staff and are therefore less able to engage with collective activity:

*‘I, on behalf of the practice, and it’s purely because of strategically when the meetings are, the collaborative meetings, the GPs that I work for, because you’re single-handed, it’s been difficult because it’s on a Thursday and we do X clinic.’*

(N290et_Practice Manager, Site E)

Although the analysis has shown that the funding for PCNs progressively increased, with deprivation being taken into account, variability across general practice was less easy to take into account. Smaller practices and those serving more deprived populations told us that they felt at a disadvantage when trying to meet the contractual requirements of the DES.

However, Site D had established an operating model that accounted for the diverse population needs, suggesting that PCNs may be able to address this over time. History and pre-existing arrangements before the introduction of PCNs had enabled them to find mechanisms to work together at scale and reduce some of the struggles that smaller practices face. A single-organisation mentality shaped decision making:

*‘… So for everything at the moment that the PCN is involved in, we do it as one organisation because we are one organisation. Whereas when I look at the way that other PCNs function, they are first and foremost a practice.’*

(N750hg-PCN GP, Site D)

The ‘one organisation’ mindset enabled them to think differently about resource allocation. Internal PCN resources were distributed based on population need rather than practice list size. This enabled resources to address some local health inequalities:

*‘I think it doesn’t because we look at what is needed in each area, so we’re able to, like I said, have the kind of health coaches in like the inner-city* [xxx] *area, and we’re able to use different resources in say* [Area Y] *or* [Area Z], *which are more affluent, and they’re needing different things. And before the pandemic we’d started doing some kind of community engagement things, so we’d got like a knitting group, and we’d got different things in each practice but, obviously, depending on what was needed.’*

(N280dy-PCN GP, Site D)

Although this way of working was discussed positively by Site D PCN members, some practices may not want to work at scale in this way, as it reduces independent practice development and requires the adoption of ways of working that work for the majority. The internal dynamics within some PCNs made this difficult, particularly where there were considerable disparities in the size of practices:

*‘This one big practice that’s always not as contributing towards it than the other, so being a large practice when they are not contributing, it has a big impact on all of the smaller practices, because at the end of the day you don’t want to … It’s like a family, isn’t it? So if there’s one member who slacks, the other has to take the pressure on, and how much you can take is the big question … and because GPs change, the leadership change, the board employees, like the managers, everybody keeps changing, it has to always have its questions on going forward, is this going to work, is this going to work? There’s always doubt.’*

(N570mu_090721_Practice Partner_ Site B3)

Working together collectively and redistributing funding internally requires mature relationships, and fostering this will be an important goal for PCNs as they develop. PCNs that have been established based on historical working relationships were at an advantage, relationships had been formed and tested, allowing for PCN governance practices to be embedded more easily.

### Policy design factors

The PCN policy acknowledges the importance of primary care working together with other community-based organisations.^14^ This is particularly important when it comes to tackling local health inequalities. However, as highlighted through the quantitative analysis, the PCN contract provides funding specifically for general practice, with wider collective activity not incentivised or paid for. In some areas this was problematic. For example, in Site D, there were well-established programmes of work that had been developed by the CCG. The primary focus of these was to tackle local inequalities, with a broader focus than just health. However, driven by the incentives in the policy, PCNs within Site D retreated from the local programmes of work to focus on meeting the requirements of the PCN DES. The PCN policy was perceived to be primary care focused, which did not necessarily allow PCNs to address local issues that had been identified:

*‘… we looked at what were the true health inequalities in each of those three places and what were… the* [programmes] *have got the answers to some of these issues … but it’s broader than the health and social care agenda, it’s about housing, it’s about education, it’s about employment, it’s about economic recovery, it’s all of those things, isn’t it.’*

(N670rd-Partnership Lead, Site D)

Over time, however, growing maturity as an organisation allowed local stakeholders to recognise that there was a local need for the community programmes to operate alongside PCNs to help address local inequalities. Although PCNs were tasked with tackling inequalities, it was recognised locally that PCNs were unable to address the broader factors that shape health and inequalities, and a single approach was inadequate to address local inequalities:

*‘And now that’s a bit more established I think as a place where recognising that we need to, we can’t take a blanket approach to addressing health inequalities. And that’s really, what allowed the* [programmes] *to relaunch and reboot.* […] *So there’s that work that we’re supporting them on at the moment.’*

(N880h7-CCG Manager, Site D)

The PCN policy took a one-size-fits-all approach, expecting all PCNs, no matter their size, location, or population they served, to deliver the same contractual requirements. Some aspects of the policy were found to be harder to implement in more deprived areas. For example, some PCNs covering deprived populations told us that they had struggled to recruit new staff to the ARRS roles:

*‘Yeah, I mean, we have managed to recruit. There’s been some areas where it’s taken a few adverts to get people in, so, we’ve been a bit persistent. But I think there are occasional, I think they’ve found a couple of areas in the more deprived areas have struggled a little bit, so have been out for advert more than once.’*

(N130iy-CCG Manager, Site A)

In some areas they tried to mitigate this by recruiting collectively. This entailed PCNs working together at supra-PCN level to develop job descriptions and go out to recruit together. The pandemic affected PCN development and slowed down the recruitment process into the ARRS roles. In many areas, this led to underspend of the available funding. To ensure that money allocated to PCNs was not lost, PCNs began to be creative about how they could use the money. In one site, there was some *‘fudging’* of the national contract, to try to meet local demand, focusing on community members more likely to suffer from health inequalities. This is an example of where a potentially restrictive contract was used a little differently to address known local need.

Community provider 1*: ‘We are having conversations with the commissioner about the ARRS underspend and where we can do something different. We are looking at a piece of work where we would prioritise the most deprived areas in* [XXX]*, including the BAME communities. We know that there people are more likely to suffer from health inequalities. This would mean that they would get bumped up the waiting list.’*

Community provider *2: ‘This would provide more opportunities to engage with the Patient Ambassadors.’* PCN business manager*: ‘It sounds positive. We know that this is a good service. It is a shame that we can’t get it closer to* [Name of] *Street.’* (PCN A1, PCN Members Meeting, 070921)

More generally, respondents reported that, in common with other incentive schemes, the targets associated with PCNs (particularly regarding the Investment and Impact Fund element of the contract) could be more difficult to meet in deprived areas. Some of the PCN targets were not new to general practice, such as flu immunisation; however, the responsibility to deliver them had changed from an individual practice one to a PCN one. There was recognition that some of these targets had been unobtainable for many years and that changing the entity that was required to deliver the targets would not necessarily make the task any easier:

*‘So we get data as a PCN, don’t we, about not achieving on X, Y, and Z target, whatever it is which relates to health, but we’re never going to achieve that because of the deprivation that we have. But yet we can’t find that information … we find it really challenging to find that information.’*

(N840im-PCN Consultant, Site A)

PCN staff serving deprived populations spoke of the unfairness that they experienced when trying to meet the targets, highlighting that PCNs and practices serving more affluent areas will find it easier, as their population was more engaged and willing to use local healthcare services. This was visible during the COVID-19 vaccination programme, with more deprived areas experiencing higher levels of vaccine hesitancy.

## Discussion

### Summary

This paper has explored the implementation of PCN policy and the factors affecting their ability to tackle health inequalities. It has identified three mechanisms by which PCNs are expected to potentially address health inequalities, and explored how these are working in practice. The funding formula was initially pro-rich relative to need; however, over subsequent iterations of the policy this relationship became more pro-poor and is now more pro-poor than the adjustment used in the general practice global sum. This is encouraging, as it suggests that the need to account for deprivation within funding allocations is understood by national policymakers.

The direct requirement for action to tackle health inequalities has been generally welcomed, but the task is felt by some to be daunting, given the importance of social factors beyond the reach of health services. PCNs have a lot of potential for collective action and the redistribution of funding between members to address inequalities. However, this depends on mature and trusting relationships, and the development of a collective mindset as well as robust internal processes.

PCN characteristics in terms of size, membership, and patient demographics are important enabling or inhibiting factors regarding addressing health inequalities, with those including multiple small practices and those serving deprived populations at particular disadvantage. There is some evidence that those serving deprived areas find it more difficult to both take advantage of PCN funding and achieve relevant targets, and some flexibility within the policy may be required.

### Strengths and limitations

The mixed-methods approach was a particular strength of the study. Initial interviews suggested some concerns about the extent to which funding for PCNs took account of deprivation, leading the authors to undertake the quantitative analysis, which showed that, over time, funding has progressively shifted to take account of measures of deprivation. The emerging findings from the quantitative work were then able to inform the ongoing data collection. This type of integrated mixed-methods research can be difficult to carry out, but it provides a rich and detailed understanding of complex and nuanced phenomena. The datasets used in the quantitative methods covered all PCNs, which allowed an analysis of how the funding formula addresses need by deprivation. The qualitative case studies used a longitudinal approach exposing the ongoing local challenges faced by PCNs. This methodology provides a voice to those who are trying to implement national policies within local contexts, illuminating challenges that may not be visible at a national level. The trustworthiness of the qualitative findings rests on the triangulation of interview data with data from observation of PCN meetings, the authors’ engagement with relevant literature before recruitment, and the collective analysis of the transcribed interviews. Individual team member’s interpretations were discussed and revised, with reflexive engagement with the positionality and experiences of each team member. However, the qualitative interviews took place within a specific timeframe and this is a limitation, as the landscape around PCNs has changed over time. Further work is required to understand how PCNs are operating outside of the COVID-19 pandemic, which significantly changed the expectations of the PCN contract. In addition, more research is required to understand how PCNs are working within newly formed integrated care systems (ICS), introduced in the Health and Care Act 2022.^27^ ICSs are statutory bodies with a responsibility for planning and delivering health and care services to their local population (an average of 1.5 million patients). Since their introduction, CCGs have been abolished and their responsibilities have been subsumed into each ICS.

The Carr-Hill formula is commonly criticised for incompletely accounting for workload differences caused by deprivation,^28^ meaning the coefficients developed here are likely to be underestimated relative to the true need of deprived communities. The CCG allocation formula provided greater weighting for income deprivation than Carr-Hill. Given the capacity and access fund uses the CCG allocation formula, the authors expect PCN funding to have remained pro-poor. A sensitivity analysis including these funding data confirmed this expectation.

Unfortunately, funding uptake by PCNs could not be analysed, as too much of the publicly available data are missing. The amount networks actually receive may vary by deprivation, particularly for the ARRS and Impact and Investment Fund, which require engagement by the network, and further analysis could usefully explore this variation.

### Comparison with existing literature

It is well known that practices in more deprived areas have struggled to engage with incentive schemes and it is unsurprising that the same issues arise with PCN incentives.^29^ Adjusting funding for the additional work associated with working with deprived populations is difficult, but can be important in mitigating the advantages associated with working in more affluent areas.^30^ More generally, evidence from previous schemes encouraging groups of GPs to work together has highlighted the importance of good management support.^31^ PCN policy at present does not include the deployment of dedicated managers, and this may represent a potential avenue through which to support all PCNs to work more closely together and develop collective approaches to inequalities.

### Implications for research and practice

The detailed study has highlighted important issues for PCNs as they seek to tackle health inequalities and has suggested four potential approaches that could be adopted to support them in this task. First, more aspects of the funding model could be weighted, alongside better adjustment of incentive scheme requirements to reflect the additional difficulties faced by PCNs serving deprived populations. Second, additional management support, both internally and at supra-PCN level, could be usefully provided, particularly for PCNs situated in deprived areas. Third, support should be provided to encourage PCNs to redistribute funds internally to help support more deprived practices. This is particularly important for PCNs that serve heterogeneous populations. Finally, for PCNs to really tackle local inequalities, what they are asked to do needs to be specific and consider what general practice can realistically achieve. It is known that inequalities are a ‘wicked’ problem, and that health care cannot effect change alone. However, PCNs represent a promising vehicle for change, and the study has suggested ways in which their potential may be realised.

## Data

Data available on request from the authors [JH].
